# A fully automatic method for segmentation of soccer playing fields

**DOI:** 10.1038/s41598-023-28658-1

**Published:** 2023-01-26

**Authors:** Carlos Cuevas, Daniel Berjón, Narciso García

**Affiliations:** Grupo de Tratamiento de Imágenes (GTI), Information Processing and Telecommunications Center (IPTC), Universidad Politécnica de Madrid (UPM), 28040 Madrid, Spain

**Keywords:** Electrical and electronic engineering, Computer science

## Abstract

This paper proposes a strategy to segment the playing field in soccer images, suitable for integration in many soccer image analysis applications. The combination of a green chromaticity-based analysis and an analysis of the chromatic distortion using full-color information, both at the pixel-level, allows segmenting the green areas of the images. Then, a fully automatic post-processing block at the region-level discards the green areas that do not belong to the playing field. The strategy has been evaluated with hundreds of annotated images from matches in several stadiums with different grass shades and light conditions. The results obtained have been of great quality in all the images, even in those with the most complex lighting conditions (e.g., high contrast between sunlit and shadowed areas). In addition, these results have improved those obtained with leading state-of-the-art playing field segmentation strategies.

## Introduction

Association football, more commonly known as football or soccer, is the most popular sport worldwide^1,2^. Since its foundation, in 1863, it has spread throughout the planet, reaching almost all countries^3^. It is the sport with the largest television audience with nearly 4 billion followers^4^, it has millions of practitioners^5^, and it is also the most studied sport^6^.

As a consequence of this great popularity and thanks to the technological advances produced in the last decade, the demand for artificial vision applications to automatically analyze soccer matches has grown enormously in recent years^7^. On the consumer end, spectators demand applications capable of enriching the content of live broadcasts^8^. Meanwhile on the professional side, clubs and players request applications aimed at a better understanding of the game, studying team tactics, or creating training sessions to improve player performance^9–11^. Additionally, the Video Assistant Referee (VAR), introduced in 2018 into the Laws of the Game of the International Football Association Board (IFAB) to help referees in reviewing decisions by means of video footage, has given rise to a notable increase in the technology used in the stadiums^12^. Besides, during recent years there has been a surge of interest in applications focused on accurately predicting soccer games^4^.

### Motivation

Many soccer-related applications require detecting different kinds of elements that appear on the playing field: players, ball, line marks, and grass bands. The detection of the players^13^ and/or the ball^14^ is used in applications that try to identify relevant events that take place during the matches (e.g., goal scoring, shots on goal, or corner kicks), or in applications that perform high-level analyses about the matches (e.g., possession statistics, offside detection, or team tactics). Regarding the detection of the line marks and the grass bands^15^, it is used in applications that require to register the images in a model of the playing field and/or estimate the pose of the cameras for high-level purposes, such as including augmented reality in images or obtaining some player statistics (e.g., distance traveled, speed, etc.).

**Figure 1:**
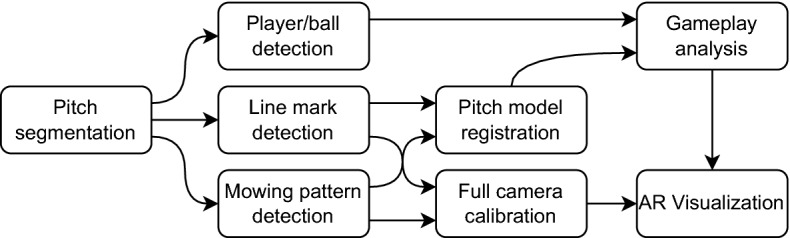
Possible system pipeline including the proposed method as a first step.

The pipeline in Fig. 1 illustrates how the proposed segmentation strategy could be used to complement the analysis strategies described in the previous paragraph. The segmentation of the playing field allows discarding areas of the images that are irrelevant for such strategies (e.g., the stands, the billboards or the sky)^7^. Therefore, included as a first stage in any of them, it has the potential to prevent false detections outside the playing field, thus improving their results.

It is important to note that all of these applications are intended for use on video streams. Consequently, the segmentation strategies that are proposed to complement them must be fully automatic.

Typical segmentation strategies assume green as the dominant color in the playing field and try to isolate such color by analyzing the histogram of the hue component in the HSV (hue, saturation, value) color space or by establishing simple rules in the RGB (red, green, blue) color space. Although these methods are capable of providing high-quality results on many images, they fail in images containing certain colors in the stands or in the billboards, and also in images featuring heavy contrast between sunlit and shadowed areas. Furthermore, all of these strategies include region-level post-processing steps that depend on thresholds which, in turn, depend on the characteristics of the images (e.g., the zoom and the spatial resolution). This scene-dependent thresholds make it difficult for strategies to be fully automatic.

### Contribution

We propose a new playing field segmentation strategy that is capable of providing very high quality results in images taken from different points of view and in complex situations (e.g., lawn striping resulting from bending grass in different directions, or images with a high contrast between sunlit and shadowed areas). Two public databases that contain over 450 manually annotated images from 25 matches in several stadiums with different shades of grass and light conditions have been used to assess the quality of our strategy. This evaluation has shown that our results are significantly better than those of most outstanding playing field segmentation methods.

The specific contributions of this paper are:

1. An analysis based on normalized green is combined with another analysis based on chromatic distortion in the RGB color space to segment, at the pixel level, the green areas of images. The combination of these two analyses allows obtaining high-quality results regardless of the shades of green of the grass bands and the lighting of the stadiums.
2. A post-processing stage at the region level that allows discarding areas of the image that do not belong to the playing field but that show a color similar to that of grass. Unlike the post-processing included in other methods, the one we propose does not depend on scene-dependent thresholds, which greatly simplifies its application regardless of the characteristics of the images (e.g., their resolution) and their content (e.g., the size of players).

### Organization

The paper is organized as follows. First, “Related work” section  reviews the main image image segmentation strategies in the literature, paying special attention to those used in soccer. In “System overview” section an overview of the proposed strategy is provided, which includes three main parts: a pre-processing stage (“Pre-processing” section), a pixel-level analysis (“Pixel-level analysis” section), and a region-level analysis (“Post-processing” section). Experiment results are reported in “Results” section and, finally, “Conclusions” section presents the conclusions of the paper.

## Related work

This section summarizes the main types of strategies that can be found in the literature to segment images. First, general purpose strategies are mentioned. Secondly, those used in the scope of soccer are described. Finally, we focus on the strategies that, like the proposed one, have the aim of segmenting the playing field in soccer images.

### Image segmentation

The goal of image segmentation is to segment an image into consistent objects or regions of interest (ROI)^16^. It is a pre-processing stage in many image-based applications like biometric identification, medical imaging, object detection and classification, and pattern recognition^17^.

In the literature, a wide range of image segmentation algorithms have been developed, starting with the simplest ones like thresholding^18^, region-growing^19^, k-means clustering^20^, or watershed methods^21^, and progressing to more complex ones like active contours^22^, graph cuts^23^, conditional and Markov Random Fields^24^, or deep learning methods^25^.

In addition, there are many different applications of video analysis that require segmenting the moving objects in the scene (called foreground), separating them from the static data (called background)^26^, such as video surveillance^27^, Human-Machine Interaction (HMI)^28^, or object tracking^29^. To achieve this, a variety of moving object segmentation algorithms have been presented in the literature, ranging from unsupervised techniques (e.g., background subtraction) to semi-supervised (e.g., spatio-temporal graphs) or interactive (e.g., graph partitioning models) techniques^30^.

### Segmentation in soccer

In the case of image analysis of soccer matches, we can find in the literature algorithms designed to carry out the following three types of segmentation:

- Playing field segmentation: They are focused on segmenting the playing field and are typically based on modeling the greenness of the grass (see “Soccer playing field segmentation” section).
- Line mark segmentation: They focus on the segmentation of the lines that delimit the playing field, since these lines are required to register the images in a model of the playing field and/or estimate the pose of the cameras, which in turn allows addressing different high-level tasks, such as including virtual elements in the images^31^.
- Players and/or ball segmentation: They try to segment the moving objects on the playing field (i.e., players and ball). These objects are typically taken as input data by different types of high-level analysis applications (e.g., analysis of the ball possession or analysis of team tactics)^7^.

### Soccer playing field segmentation

Although it is technically possible to segment the playing field using many of the segmentation algorithms mentioned above (e.g., watershed, region-growing, or deep learning), due to their simplicity and because the green color of the grass is the most significant feature of the playing field, the most outstanding strategies in the literature use thresholding algorithms that are applied to histograms of different color components. These strategies can be classified into the following three types:

- Hue-based methods: Those based on the hue component of the HSV color space.
- RGB-based methods: Those based on the analysis of the RGB color space.
- *g*-based methods: Those that analyze the green chromaticity (*g*) of the image.

Hue-based methods analyze the histogram of the hue component in search of the dominant mode, which is presumed to correspond to the green color of the playing field. Then, they separate this mode from the rest of the histogram data. In some works^32,33^, fixed thresholds around the dominant mode in the hue histogram are used. In^34^, the thresholds are selected by considering a fixed width around the dominant mode of the hue histogram. In^35,36^, training sets of images are used to determine the average position and width of the dominant peak in the hue histograms. Although hue-based methods are able of providing successful results in simple cases, automatically selecting the appropriate thresholds can be very complex in some images, as hue histograms are often very multimodal. Moreover, these methods are very sensitive to the size of the histogram bins and fail in images where the dominant mode does not correspond to the color of the playing field, but to the sky or the stands^31^.

RGB-based methods, assuming that the color of the playing field is primarily green, try to segment the playing field by determining what pixels satisfy certain relationships between the red (R), green (G), and blue (B) components of the RGB color space. In^37–39^, it is assumed that the pixels of the playing field must comply with the rule $G>R>B$. Alternatively, in^40^ it is assumed that the playing field pixels are those satisfying $G>R$ and $G>B$. In^41^, 2-dimensional histograms in a normalized RG color space are used to deal with grass color variations, where the peaks determine the playing field data. In^42,43^, RGB 3-dimensional histogram techniques are proposed. The main advantage of these methods is their simplicity. However, they fail in images with areas saturated by sunlight or with strong shadows.

$g$-based methods have been proposed recently as an alternative to the two types of previous methods. They start from the fact that $g$ is highly invariant to changes in illumination and, additionally, provides a very simple criterion for classifying colors in terms of their closeness to green (a purer green as $g$ increases). In^44^, the probability density function (pdf) of $g$ is approximated using a Gaussian Mixture Model (GMM). Then, the playing field mask is obtained by selecting the modes of the pdf that denote data with mainly green information and applying a region based analysis. In^45^, a similar analysis of the pdf of $g$ is applied to perform a segmentation that is robust to both floodlighting and natural lighting. Although these methods yield good-quality results in playing fields with different shades of grass and variations in lighting, they generally include areas of the billboards or stands with colors such as cyan or yellow, since the criterion they use to segment does not allow separating green from those colors.

It is important to point out that all these strategies are based on the analysis of the color characteristics of the images at the pixel level. Consequently, they require applying different kinds of region-based post-processing (e.g., morphological operations, convex hulling, etc.) to: i) discard areas of the image that do not belong to the playing field but that show a color similar to that of grass and; ii) deliver a single connected region. These post-processing stages include several thresholds that are highly dependent on the size and shape of the non-green objects that appear on the playing field (the white lines and the players), which in turn are dependent on the size of the images, the location of the camera, and the zoom used. This ad-hoc tailoring significantly reduces the usability of all these strategies.

## System overview

**Figure 2:**
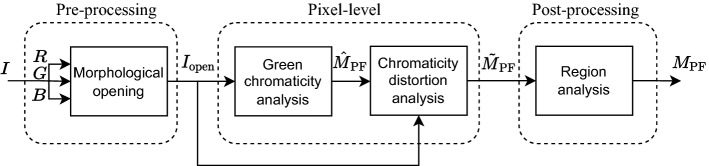
Block diagram of the proposed strategy.

The proposed strategy comprises a pre-processing block, two pixel-level processing steps, and one region-level post-processing step, as shown in Fig. 2.

In the pre-processing block (“Pre-processing” section) an RGB image $I_{\textrm{open}}$, in which the white line marks in the playing field are integrated in the grass, is derived from the original image, $I$.

Then, a pixel-level segmentation that is based on the analysis of the green chromaticity of $I_{\textrm{open}}$ is performed (“Pixel-level analysis” section), which yields an initial binary mask, $\hat{M}_{\textrm{PF}}$, indicating the pixels in which the green component is the dominant one. RGB-based chromaticity distortion analysis is then performed to remove pixels that, despite having a significant contribution from the green channel, are not part of the playing field (“Pixel-level analysis” section). As a result of this second analysis, the binary mask $\tilde{M}_{\textrm{PF}}$ is obtained.

The final mask of the playing field, $M_{\textrm{PF}}$, is obtained by applying a region-level post-processing module that discards green regions that, despite having a color compatible with the grass of a soccer playing field, are not part of it (“Post-processing” section).

## Pre-processing

In many images, the presence of white line marks results in the playing field being represented by multiple connected regions after segmentation at the pixel level. Since the number and characteristics of these regions are unknown, complex threshold-based rules are typically required at the region-level post-processing stage. The purpose of this pre-processing is to prepare the original image in such a way that after pixel-level segmentation is applied, the playing field is represented by a single connected region, thus making much easier the post-processing at the region level.

**Figure 3:**
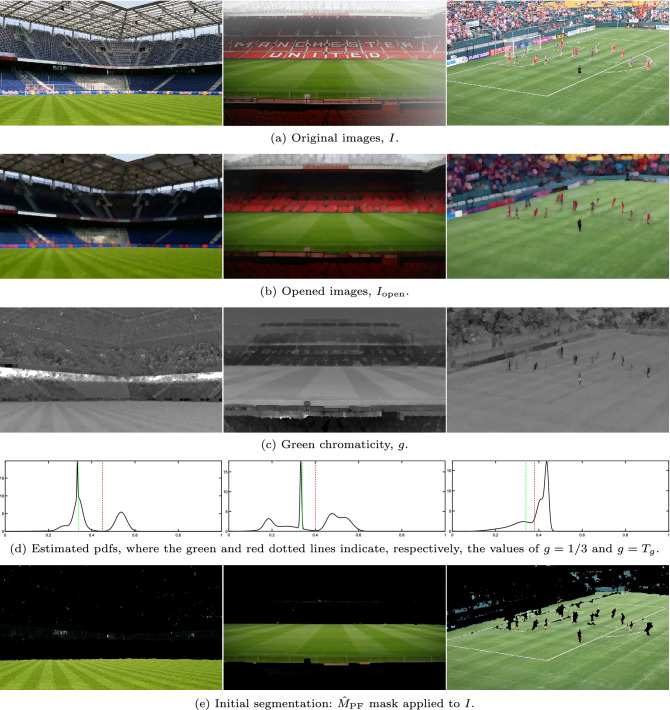
Results obtained on three images after applying the pre-processing and the analysis at the pixel level. Original images from^46,47^, and^48^.

Let $I$ be an original RGB image of a soccer match (see Fig. 3.a). To integrate the white line marks in the playing field (i.e., so that the lines adopt a color similar to that of the grass that surrounds them), a morphological grayscale opening operation is applied to each channel to obtain a new RGB image, $I_{\textrm{open}}$ (Fig. 3.b). For this, a circular flat structuring element is used, whose diameter $d_{\textrm{e}}$ must be slightly greater than the width of the line marks. If this diameter is too small, the lines will not blend well enough into the playing field. On the other hand, if it is too large, some elements of the playing field perimeter will be wrongly integrated into the grass, which will reduce the accuracy of the results. Since the thickness of the white line marks is proportional to the size of the images, we have decided to use a diameter proportional to the size of the diagonal of the image:

1 $$\begin{aligned} d_{\textrm{e}}=\frac{\alpha _{e}}{100}\sqrt{H^{2}+W^{2}}, \end{aligned}$$

where *H* and *W* are, respectively, the height and width of the image in pixels. The choice of the appropriate value of $\alpha _{e}$ and its influence on the quality of the proposed strategy are discussed in the “Results” section.

## Pixel-level analysis

### Green chromaticity analysis

This analysis makes it possible to identify, at the pixel level, the areas of the image with a greater contribution of green than other primary colors. In addition, it allows these areas to be separated based on the shade of green that characterizes them. To do this, the normalized green component of $I_{\textrm{open}}$ is analyzed, which is obtained as:

2 $$\begin{aligned} g\left( r,c\right) =\frac{G_{\textrm{open}}\left( r,c\right) }{R_{\textrm{open}}\left( r,c\right) +G_{\textrm{open}}\left( r,c\right) +B_{\textrm{open}}\left( r,c\right) }, \end{aligned}$$

where $R_{\textrm{open}}$, $G_{\textrm{open}}$, and $B_{\textrm{open}}$ are, respectively, the red, green, and blue components of $I_{\textrm{open}}$, and $\left( r,c\right)$ are the coordinates of the pixels (row and column, respectively).

The pdf of $g$ is expected to exhibit one (or several) modes corresponding to the different shades of green at high values, and several spurious modes corresponding to other colors at lower values^44^. To identify the different groups of pixels with different shades of green, first, the pdf of $g$ is approximated using the Expectation-Maximization (E-M) algorithm from a different number of Gaussian distributions (from 1 to $N_{\textrm{G}}$) initialized with equal weights and with means uniformly distributed over the data range. Then, the Akaike information criterion (AIC)^49^ is used to select the set of Gaussians that provide the best fitting. The experiments carried out have shown that $N_{\textrm{G}}=6$ is enough to obtain good fits in all the images and that, in addition, the quality obtained hardly changes if other values close to the selected one are used. The results section analyzes in detail the influence of this parameter on the strategy.

Assuming that green is the dominant color of the playing field (i.e., $G_{\textrm{open}}\left( r,c\right) >R_{\textrm{open}}\left( r,c\right)$ and $G_{\textrm{open}}\left( r,c\right) >B_{\textrm{open}}\left( r,c\right)$), the pixels that constitute it must meet $g>1/3$. Taking this into account, an initial mask of the playing field, $\hat{M}_{\textrm{PF}}$, can be easily obtained by selecting the pixels with values of $g$ greater than the threshold

3 $$\begin{aligned} T_{g}=\max \left( \frac{1}{3},m_{0}\right) , \end{aligned}$$

where $m_{0}$ is the first local minimum of the pdf that is below the first of the peaks above $g=1/3$. In this way, only the data represented by modes of the pdf whose peaks are located above $g=1/3$ are selected.

Figure 3 shows, for some representative images, their green chromaticity components (Fig. 3c), the pdf resulting from the adjustment with the E-M algorithm (Fig. 3d) and the images segmented using the mask $\hat{M}_{\textrm{PF}}$ (Fig. 3e). In the case of the first two examples (images in the left and central columns), it can be seen that the result of the segmentation is of high quality, having correctly identified the green areas of the playing field. However, in these results it can be seen that some small regions in the stands have also been segmented. These regions will be removed by applying the post-processing described in “Post-processing” section. Regarding the third of the examples in the figure (images in the right column), the playing field has also been segmented correctly. However, much of the billboards also appear incorrectly segmented as part of the grass. This is because the green chromaticity of some colors like yellow or cyan is also greater than 1/3. Therefore, green chromaticity analysis cannot discriminate between green and these other colors. To deal with this problem, the chromatic distortion analysis described below is applied.

### Chromatic distortion analysis

In the pdf obtained in the previous analysis, each of the modes above the selected threshold $T_{g}$ represents data with a similar level of green. However, as just discussed, there are some pixels that do not have the color of grass and yet are also represented by these modes (e.g., those with cyan or yellow colors). By analyzing the chromatic distortion between the RGB value of the data of each mode and the average RGB value of said data it is possible to identify and discard pixels that, having values of $g$ greater than 1/3, are nevertheless of a color very different from the green of the grass.

**Figure 4:**
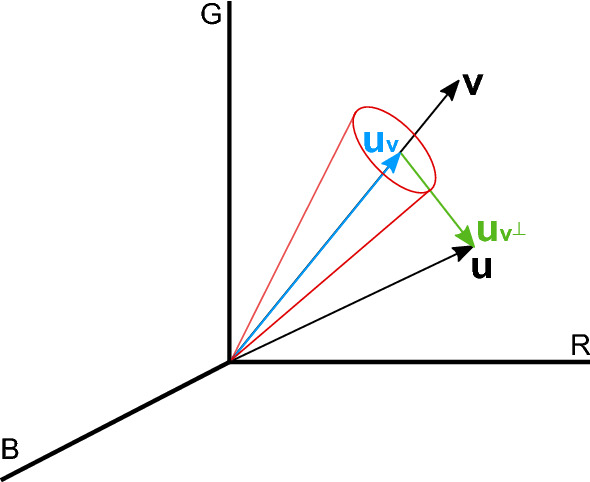
Three-dimensional representation of an example of the cone that restricts the acceptable colors given the vectors $\textbf{v}$ and ${\textbf{u}}$.

This analysis is carried out as follows:

1. Within the envelope of the set of Gaussian distributions used to estimate the pdf of $g$, the set of $N_{L}$ local minima above $T_{g}$ are selected, $L=\left\{ m_{g}\right\} _{i=1}^{N_{L}}$.
2. $N_{v}=N_{L}+2$ sets of RGB data are obtained by selecting the RGB values of the pixels of $I_{\textrm{open}}$ with values of $g$ being between:
  $T_{g}$ and the first element in *L*.
  Consecutive pairs of local minima in *L*.
  The last local minimum in *L* and 1.
3. For each of these sets, each containing RGB data with a similar green chromaticity, the chromatic distortion is analyzed to discard colors that, although similar in their green chromaticity, can be readily told apart using the full color information. Let $\textbf{v}\in {\mathbb {R}}^{3}$ be the vector with components R, G and B, representing the reference color resulting from averaging all RGB colors in the data set. All the vectors of the form $\alpha {\textbf{v}}$ represent the same color with varying luminance proportional to their magnitude, while different colors are represented by vectors running in different directions: the greater the angle between two such vectors, the more dissimilar they are. Let ${\textbf{u}}\in {\mathbb {R}}^{3}$ be the vector corresponding to the RGB color of a pixel we want to compare to the reference color ${\textbf{v}}$. We can uniquely write ${\textbf{u}}$ as the sum of two orthogonal components: ${\textbf{u}}_{\textbf{v}}=\left( {\textbf{u}}\cdot {\textbf{v}}\right) {\textbf{v}}/\left\| {\textbf{v}}\right\| ^{2}$, parallel to ${\textbf{v}}$, and ${\textbf{u}}_{{\textbf{v}}^{\perp }}={\textbf{u}}-{\textbf{u}}_{{\textbf{v}}}$, perpendicular to ${\textbf{v}}$. Thus, we can define the tangent of the angle between ${\textbf{u}}$ and ${\textbf{v}}$ as
  4 $$\begin{aligned} \textrm{cd}=\frac{\left\| {\textbf{u}}_{{\textbf{v}}^{\perp }}\right\| }{\left\| {\textbf{u}}_{{\textbf{v}}}\right\| }. \end{aligned}$$
  The greater cd, the more dissimilar ${\textbf{u}}$ and ${\textbf{v}}$, discounting the effect of lightness due to illumination changes.
4. A new mask of the playing field is obtained as:
  5 $$\begin{aligned} \tilde{M}_{\textrm{PF}}\left( r,c\right) ={\left\{ \begin{array}{ll} 1, &{} \hat{M}_{\textrm{PF}}\left( r,c\right) =1\,\wedge \,\mathrm {cd}\left( r,c\right) <T_{\textrm{c}}\\ 0, &{} \textrm{otherwise} \end{array}\right. }, \end{aligned}$$
  where $T_{\textrm{c}}$ is a threshold that effectively restricts acceptable colors to those inside a cone whose axis is the reference color, as illustrated in Fig. 4. The range of adequate values for this threshold is analyzed in the “Results” section.

**Figure 5:**
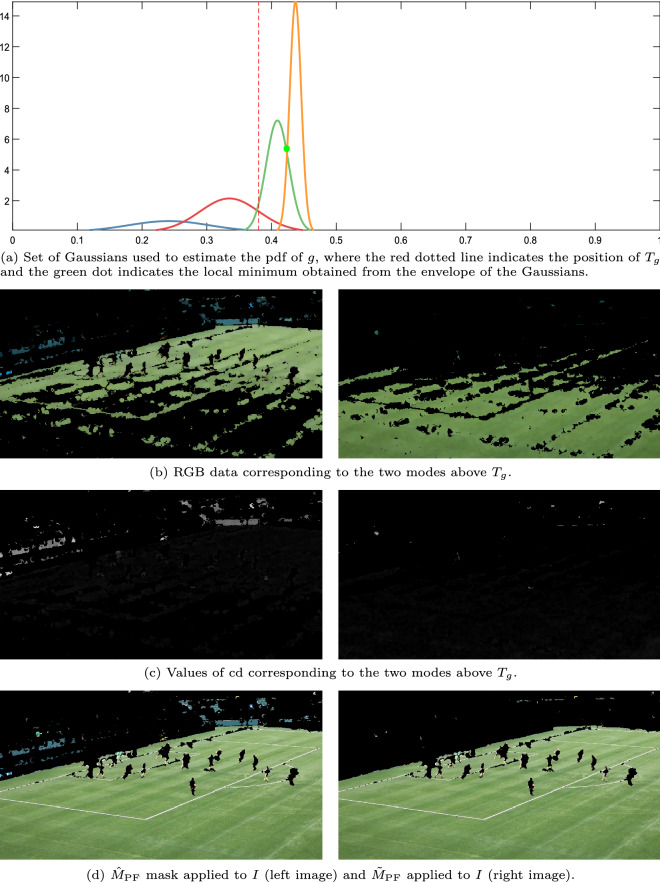
Example of the quality improvement obtained with the chromatic distortion analysis. Results corresponding to the original image from^48^.

In the example of Fig. 5, which corresponds to the third case illustrated in Fig. 3 (image in which the chromaticity analysis of green is not sufficient to correctly segment the playing field), the pdf of $g$ (Fig. 5a) has been obtained by mixing 4 Gaussian distributions, two of them representing modes above $T_{g}$. The local minima separating these two modes has been depicted with a green dot and the dotted red line indicate the position of $T_{g}$. The images in Fig. 5b,c correspond, respectively, to the RGB data and their ratios in each mode. It can be seen that billboards have given rise to the highest $\textrm{cd}$ values. Consequently, as can be seen in Fig. 5d, where the result of the segmentation is compared before and after the chromatic distortion analysis, billboards have been removed by applying Eq. (5).

**Figure 6:**
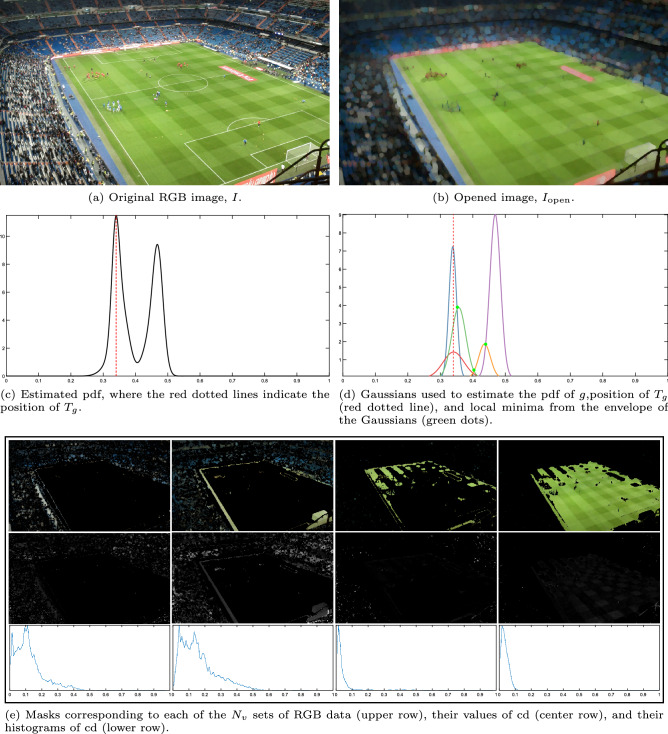
Analysis of the histograms of the chromatic distortion values. Original image from^46^.

In images where the predominant color in the stands is yellow or cyan, some of the $N_{v}$ sets of data analyzed could be composed entirely of non-pitch pixels. An example of this can be seen in Fig. 6, where the people in the stands wear predominantly yellow, and the first set of data analyzed is representing mainly pixels in the stands. To deal with this issue, before applying Eq. (5), the histogram of $\textrm{cd}$ values is analyzed. While the histograms of the data sets that are part of the playing field have a very narrow main mode that is close to zero (i.e., the RGB color of the vast majority of this data is very uniform), histograms of data sets that do not represent the playing field have a much wider main mode that, sometimes, is placed significantly away from zero (i.e. the RGB colors of the set differ considerably). Therefore, these unwanted data sets can be easily identified and discarded by setting as valid only data sets that have a histogram of $\textrm{cd}$ with a main mode below $T_{\textrm{c}}$. In the example in Fig. 6 the first two data sets analyzed have histograms that do not meet this condition. Therefore, these two data sets will be discarded.

## Post-processing

By means of the analyses described in “Pixel-level analysis” section it is possible to correctly segment the grass of the playing field. However, as these analyses are at the pixel level, the results obtained include some false detections due to elements that do not belong to the playing field but have a very similar color. Furthermore, players were also not included in the segmentation result, appearing as holes in the playing field mask. To solve these problems, the proposed strategy includes a final analysis at the region level. This analysis, unlike other segmentation strategies, is completely automatic and avoids the use of morphological operations, which largely depend on the size of the images, the location of the camera, and the zoom used.

The final binary mask, $M_{\textrm{PF}}$, resulting from this region-level post-processing is obtained as follows:

1. To include the pixels occupied by the players in the final segmentation, a flood-fill operation^50^ is applied to the mask $\tilde{M}_{\textrm{PF}}$.
2. Finally, to eliminate false detections, the largest region from the resulting ones is selected.

It is important to note that this simple mechanism to get rid of false detections is made possible by the pre-processing stage included in our strategy. If this pre-processing is not included, the mask $\tilde{M}_{\textrm{PF}}$ could contain multiple regions associated with the playing field and, therefore, it would be necessary to analyze how many regions to select.

## Results

This section summarizes the results provided by the proposed playing field segmentation strategy. First, “Analysis of the results obtained” section analyzes the quality of the results obtained, as well as the influence on this quality of each of the stages that make up the strategy. Then, “Parameter analysis” section discusses parameter selection for optimal results. “Limitations” section discusses the limitations of the strategy. Finally, “Comparison with other strategies” section compares our results with those obtained using other segmentation strategies.

To analyze the quality of our strategy, images from the following two public databases have been used, which, to our knowledge, are the only ones that provide ground truth files that include binary masks indicating what areas of the images correspond to the playing field:

- LaSoDa: The Labeled Soccer Database (LaSoDa) consists of 60 annotated Full HD images ($1920\times 1080$ pixels) corresponding to five matches played in stadiums with different characteristics (different camera positions and different shades of grass). These images show different zoom levels (from images that show only the goal area to images that show more than half of the pitch) and have been acquired with four different types of cameras (master camera, side camera, end camera, and aerial camera). Additionally, it includes challenging lighting conditions (day and night matches and strong contrast between sunlit and shaded areas). This dataset is available at https://www.gti.ssr.upm.es/data/lasoda.
- Homayounfar’s database: The database proposed in^51^ is composed by 395 HD images ($1280\times 720$ pixels) from twenty matches in stadiums with different grass textures and lighting conditions. Unlike LaSoDa, all of its images have been acquired with the master camera (the one used most of the time in soccer broadcasting, placed approximately on the extension of the halfway line) and show similar zoom levels. However, they are more varied than the LaSoDa images in terms of shades of grass and presence of shadows.

Quality has been measured at the pixel level by the recall ($\textrm{rec}$), precision ($\textrm{pre}$), and F-score ($f$) as follows:

6 $$\begin{aligned} \textrm{rec}=\frac{\textrm{tp}}{\textrm{tp}+\textrm{fn}},\;\textrm{pre}=\frac{\textrm{tp}}{\textrm{tp}+\textrm{fp}},\;f=\frac{2\textrm{tp}}{2\textrm{tp}+\textrm{fp}+\textrm{fn}}, \end{aligned}$$

where $\textrm{tp}$, $\textrm{fn}$, and $\textrm{fp}$ are, respectively, the amounts of true positives, false negatives and false positives. Note that the F-score is also known as F1-score or Dice Similarity Coefficient (DSC).

Regarding the computational cost of the strategy, the most costly step, by far, is the well-known E-M algorithm that is used to approximate the pdf of $g$. However, the literature reports that it is feasible to run E-M on a problem of our scale (histograms made of just a few hundred data points) within very few milliseconds^52^. Consequently, we consider it feasible to make our system work in real time on video sequences.

### Analysis of the results obtained

Table 1 summarizes the results obtained for each of the 25 matches in which the 455 analyzed test images are distributed (the images corresponding to all these results are available at https://www.gti.ssr.upm.es/data/playing-field-segmentation). These results correspond to the following cases:

- Case 1: Results from the mask $\hat{M}_{\textrm{PF}}$ (after performing the green chromaticity analysis).
- Case 2: Results from the mask $\tilde{M}_{\textrm{PF}}$ (after performing the chromatic distortion analysis).
- Case 3: Results from the mask $M_{\textrm{PF}}$ (final results).

**Figure 7:**
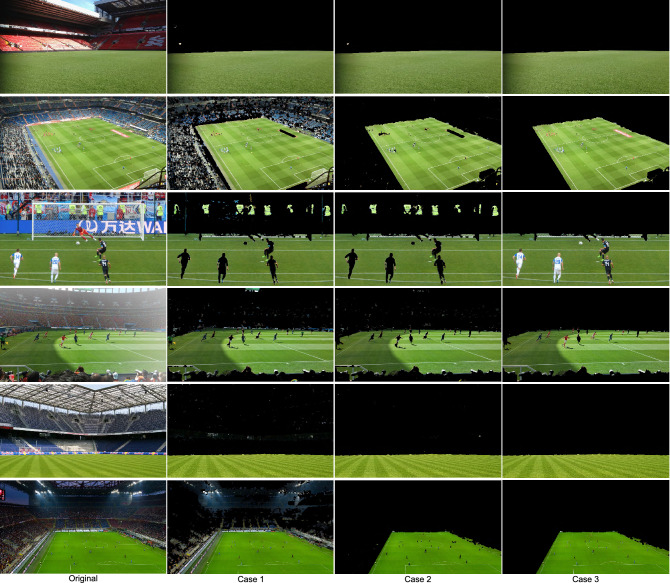
Some representative results obtained with the proposed strategy. Original images from^46^ and^53^.

In addition, Fig. 7 shows some representative results obtained in images with different lighting conditions, shades of grass, zoom levels and colors on billboards and stands.

**Table 1:** Summary of results obtained with the proposed strategy.

Match	Frames	Case 1			Case 2			Case 3		
		$\textrm{rec}$	$\textrm{pre}$	$f$	$\textrm{rec}$	$\textrm{pre}$	$f$	$\textrm{rec}$	$\textrm{pre}$	$f$
LaSoDa										
1	12	0.984	0.865	0.920	0.970	0.937	0.953	0.988	0.990	0.989
2	12	0.993	0.974	0.984	0.978	0.985	0.982	0.992	0.997	0.994
3	12	0.986	0.964	0.975	0.983	0.973	0.978	0.994	0.988	0.991
4	12	0.991	0.923	0.956	0.975	0.978	0.976	0.989	0.995	0.992
5	12	0.980	0.935	0.957	0.963	0.981	0.972	0.982	0.987	0.984
Homayounfar										
1	22	0.996	0.661	0.795	0.959	0.971	0.965	0.983	0.996	0.989
2	14	0.988	0.893	0.938	0.959	0.976	0.967	0.977	0.993	0.985
3	15	0.986	0.852	0.914	0.969	0.987	0.978	0.986	0.998	0.992
4	17	0.998	0.756	0.860	0.963	0.987	0.975	0.981	0.997	0.989
5	16	0.980	0.942	0.960	0.970	0.985	0.977	0.987	0.988	0.987
6	17	0.961	0.929	0.944	0.951	0.980	0.966	0.976	0.999	0.987
7	11	0.978	0.960	0.969	0.973	0.994	0.984	0.983	1.000	0.991
8	29	0.997	0.722	0.837	0.964	0.988	0.976	0.981	0.996	0.988
9	33	0.996	0.760	0.862	0.955	0.987	0.971	0.971	0.999	0.985
10	12	0.976	0.966	0.971	0.966	0.992	0.979	0.986	0.998	0.992
11	15	0.986	0.905	0.944	0.973	0.993	0.983	0.989	0.998	0.994
12	23	0.990	0.875	0.929	0.970	0.993	0.982	0.985	0.999	0.992
13	15	0.990	0.923	0.955	0.971	0.991	0.981	0.983	0.994	0.988
14	15	0.983	0.941	0.962	0.973	0.990	0.981	0.988	0.998	0.993
15	20	0.995	0.848	0.916	0.971	0.986	0.978	0.988	0.995	0.992
16	42	0.987	0.833	0.903	0.958	0.978	0.968	0.978	0.998	0.988
17	22	0.983	0.956	0.969	0.971	0.991	0.981	0.988	0.999	0.993
18	18	0.975	0.938	0.956	0.962	0.990	0.976	0.986	0.997	0.991
19	22	0.997	0.735	0.846	0.954	0.985	0.969	0.975	0.999	0.987
20	17	0.989	0.866	0.923	0.969	0.982	0.975	0.989	0.993	0.991
Overall	455	0.988	0.860	0.919	0.967	0.982	0.974	0.984	0.995	0.990

The high recall values obtained after applying the green chromaticity analysis (Case 1) shows that this first stage of analysis correctly identifies the vast majority of the pixels that make up the playing field. However, false detections due to the presence of cyan or yellow regions have resulted in significantly lower precision values, especially in the case of the images of some matches (e.g., Match 3) in Homayounfar’s database in which the predominant color in the stands is yellow or cyan.

Most of these false detections disappear after applying the chromatic distortion analysis (Case 2), which results in a significant increase in precision.

The final results (Case 3) show that, after applying the analysis at the region level, an improvement in both recall and precision is achieved. This is because the gaps due to the presence of players on the playing field have been filled in and, in addition, the false detections caused by small regions in the stands with colors similar to those of the grass have been eliminated.

### Parameter analysis

We had previously stated that the strategy depends on three parameters that must be configured manually (one in the pre-processing stage and two in the pixel-level analysis stage). In this subsection, the influence of these parameters on the quality of the results is analyzed.

**Figure 8:**
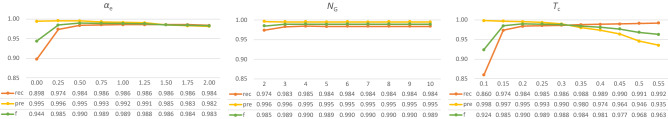
Quality of the results based on the values of the parameters on which the proposed strategy depends.

The results in Table 1 have been obtained with the combination of parameters that has resulted in the highest overall F-score. These parameters are summarized in Table 2, whereas the graphs in Fig. 8 report the variations in quality of the results when any one of them is modified. The following conclusions can be obtained from these graphs:

- Proportionality factor that determines the diameter of the structuring element used in the pre-processing, $\alpha _{e}$: Although the best results are obtained with $\alpha _{e}=0.5$, for higher values of this parameter the quality is only very slightly reduced. On the other hand, if $\alpha _{e}$ is too low (e.g., $\alpha _{e}=0.25$) or the pre-processing is not applied (i.e., $\alpha _{e}=0$), the quality reduction is very noticeable, since the white lines are not well integrated into the grass.
- Maximum number of Gaussian distributions in the estimation of the pdf of the green chromaticity, $N_{\textrm{G}}$: For values above 2 the quality is very similar, being slightly better in the case of $N_{\textrm{G}}=6$.
- Maximum allowed chromatic distortion, $T_{\textrm{c}}$: The quality of the results is very high with values of $T_{\textrm{c}}$ in a relatively wide band ($T_{\textrm{c}}\in \left[ 0.15, 0.4\right]$). Outside this range the quality is noticeably reduced.

**Table 2:** Set of parameters used in the reported results.

$\alpha _{e}$	$N_{\textrm{G}}$	$T_{\textrm{c}}$
0.5	6	0.2

This analysis shows that none of the parameters is especially critical for the strategy, since all of them have significantly wide ranges of values in which the quality of the results is very similar.

### Limitations

It should be noted that the proposed segmentation strategy is based on the assumption that the playing field is the largest green element in the image.

Consequently, it can fail in scenarios where the playing field is surrounded by large regions that are also green.

Although these situations are not common in professional stadiums (there is usually a wide variety of colors in the stands due to the amount of spectators that occupies them), they can occur in stadiums with green stands and with little or no fans, or in non-professional playing fields that, instead of being surrounded by stands, are surrounded by vegetation.

**Figure 9:**
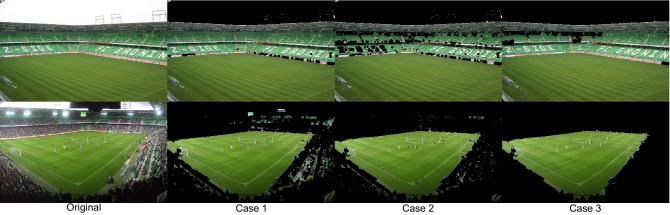
Results obtained in a stadium with green stands: without an audience (top row of images) and with some audience (bottom row of images). Original images from^54^ and^55^.

An example of this limitation is illustrated in Fig. 9, where the top row of images show the results obtained in a stadium with empty green stands and the bottom row of images shows the results in the same stadium but with a large number of spectators in the stands.

### Comparison with other strategies

The proposed strategy has been compared to four playing field segmentation methods that are representative of the three types of strategies described in “Related work” section:

- M1: RGB-based method used in^37–39^, which is based in the rule $G>R>B$.
- M2: RGB-based method recently proposed in^40^, which uses the rules $G>R$ and $G>R$.
- M3: Hue-based method used in the strategies in^32–36^, which is based on separating the dominant mode from the rest of the data in the histogram of the hue component.
- M4: *g*-based method used in^31,44^, which is based on the analysis of the pdf of the green chromaticity.

The comparisons have been made both in the case of not applying any post-processing at the region level (called Case 2 in “Analysis of the results obtained” section) and applying post-processing (Case 3). As stated in “Related work” section, the strategies in which these methods are used apply different region-based post-processing stages. To make a fair comparison between methods and given that the post-processing in the proposed strategy is the only one that does not depend on pre-established thresholds, our post-processing has been applied to all methods in the evaluation of the Case 3.

Since the area of the playing field visible in any image is always a single convex region, many of the strategies we compare against apply a convex hull as the last stage of their region-level post-processing. For this reason, we have decided to include in the comparisons a fourth case (Case 4) in which convex hulling is applied as the last stage of the post-processing.

**Figure 10:**
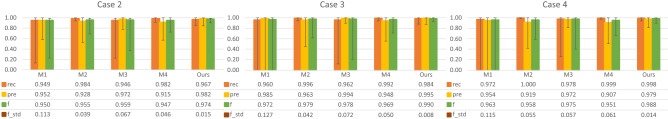
Summary of the quality obtained with our strategy and 4 other segmentation methods. The error bars report the best and worst result for each metric across the whole dataset, showing the robustness of our proposal.

The graphs in Fig. 10 compare the global quality obtained with our strategy and with the 4 previously described methods in the three cases mentioned. In these graphs, in addition to the values of $\textrm{rec}$, $\textrm{pre}$, and $f$, the range of values of each of these variables has also been included, as well as the standard deviation of the values of $f$ ($f_{\textrm{std}}$).

**Figure 11:**
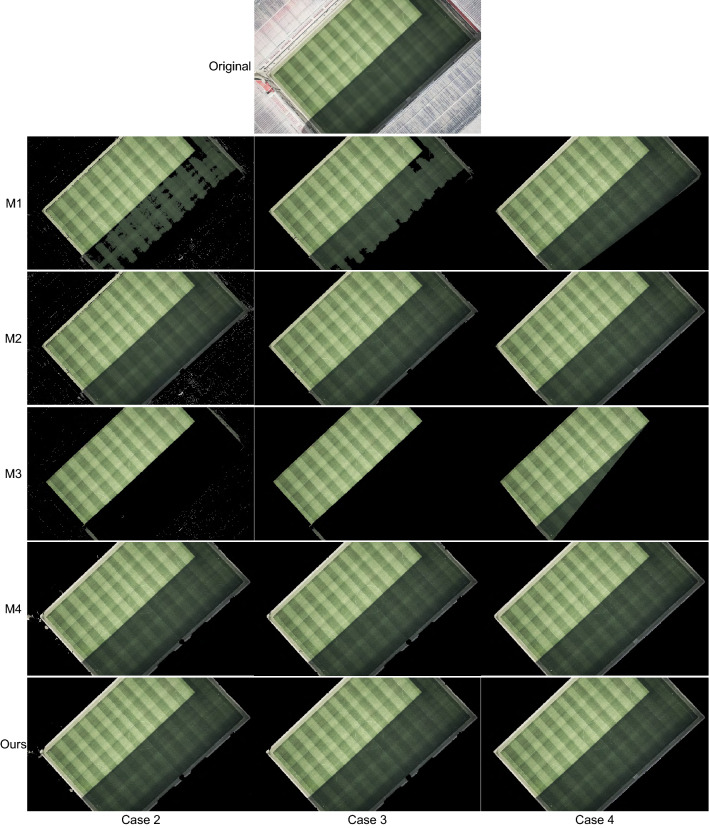
Results obtained in an image with strong shadows. Original image from^46^.

**Figure 12:**
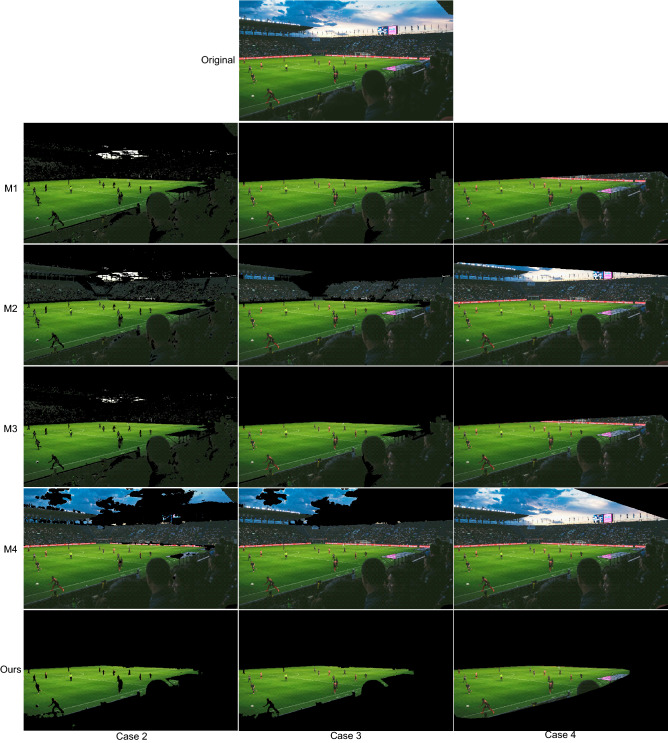
Results obtained in an image with poor color information in the stands. Original image from^46^.

**Figure 13:**
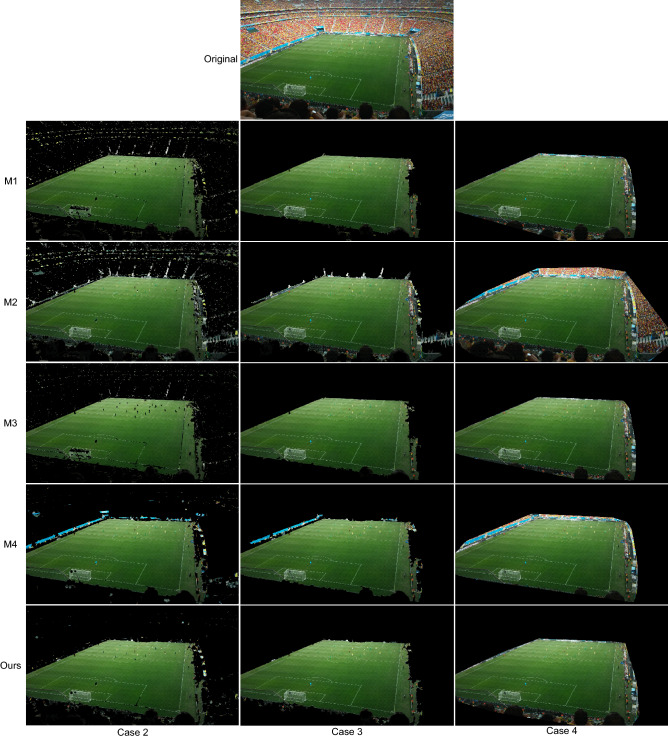
Results obtained in an image with cyan billboards. Original image from^46^.

The results before applying the post-processing (Case 2) show that our strategy is the one that obtains the best results overall. The methods M1 and M3 result in many false negatives in images with areas of the playing field with strong shadows (see Fig. 11). In images where the stands include areas with poor color information (i.e. the red, green, and blue channels are very similar) the methods M1 and M2 result in several false positives (see Fig. 12). Regarding, the method M4, it fails in images with areas with colors that cannot be correctly filtered in the green chromaticity color space (e.g., the sky in Fig. 12 or the billboards in Fig. 13).

The graphs in Fig. 10 also show that after including post-processing (Case 3) the quality of the results of the five compared strategies is improved (with our strategy still obtaining the best results).

Regarding the Case 4, as expected, by including the convex hulling in the post-processing the recall of all methods is improved. However, this improvement does not compensate for the worsening of the precision values (i.e., the F-score values get worse). The method least affected by this quality reduction is the one proposed.

Finally, we must take into account that our strategy is not only the one that provides the best overall results (the highest F-score), but it is also the one that provides the lowest value of $f_{\textrm{std}}$, which shows that our results are the most consistent.

## Conclusions

A novel strategy to efficiently segment the playing field in soccer images has been presented. By approximating the pdf of the green chromaticity using the E-M algorithm, the pixels of the image that mainly include green data are segmented. Then, to remove the pixels that are not part of the grass but have colors with an important contribution of green (e.g., yellow), a chromatic distortion analysis is performed. Finally, a region-level stage that does not depend on any user-defined parameter allows removing regions that do not belong to the playing field but that show a color similar to that of grass.

This strategy has been validated on two databases that feature a wide variety of stadiums with different shades of grass and illumination conditions, yielding excellent results with a single set of parameters across both databases and clearly outperforming existing state-of-the-art approaches.

## Data Availability

All the original images and their corresponding results are available at https://www.gti.ssr.upm.es/data/playing-field-segmentation.

## References

[1] Khodaee, M. & Mathern, S.A. Soccer. In: *Sports-Related Fractures, Dislocations and Trauma* 951–953. 10.1007/978-3-030-36790-9 (2020).

[2] Lucas GM, Gratch J, Malandrakis N, Szablowski E, Fessler E, Nichols J (2017). Goaalll!: Using sentiment in the world cup to explore theories of emotion. Image Vis. Comput..

[3] Tovar J (2021). Soccer, world war II and coronavirus: A comparative analysis of how the sport shut down. Soccer Soc..

[4] Eryarsoy, E. & Delen, D. Predicting the outcome of a football game: A comparative analysis of single and ensemble analytics methods. In: *Proceedings of the 52nd Hawaii International Conference on System Sciences* 1107–1115. 10.24251/HICSS.2019.136 (2019).

[5] Félix, L. G. S., Barbosa, C. M., Carvalho, I. A., da F. Vieira, V. & Xavier, C. R. Forecasting soccer market tendencies using link prediction. In: *Computational Science and Its Applications—ICCSA 2020*, 663–675. 10.1007/978-3-030-58799-4_48 (2020).

[6] Kirkendall DT (2020). Evolution of soccer as a research topic. Prog. Cardiovasc. Dis..

[7] Cuevas C, Quilon D, Garcia N (2020). Techniques and applications for soccer video analysis: A survey. Multimed. Tools Appl..

[8] Goebert C, Greenhalgh GP (2020). A new reality: Fan perceptions of augmented reality readiness in sport marketing. Comput. Hum. Behav..

[9] Andrienko G, Andrienko N, Anzer G, Bauer P, Budziak G, Fuchs G, Hecker D, Weber H, Wrobel S (2021). Constructing spaces and times for tactical analysis in football. IEEE Trans. Visual Comput. Graph..

[10] Narizuka T, Yamazaki Y, Takizawa K (2021). Space evaluation in football games via field weighting based on tracking data. Sci. Rep..

[11] Pons E, García-Calvo T, Cos F, Resta R, Blanco H, López del Campo R, Díaz-García J, Pulido-González JJ (2021). Integrating video tracking and GPS to quantify accelerations and decelerations in elite soccer. Sci. Rep..

[12] Armenteros, M., Benitez, A. J. & Betancor, M. Á. *The Use of Video Technologies in Refereeing Football and Other Sports*. 10.4324/9780429455551-8 (2019).

[13] Caetano FG, Barbon-Junior S, Torres RdS, Cunha SA, Ruffino PRC, Martins LEB, Moura FA (2021). Football player dominant region determined by a novel model based on instantaneous kinematics variables. Sci. Rep..

[14] Rezaei A, Wu LC (2022). Automated soccer head impact exposure tracking using video and deep learning. Sci. Rep..

[15] Cuevas C, Berjón D, García N (2022). Grass band detection in soccer images for improved image registration. Signal Process. Image Commun..

[16] Mittal H, Pandey AC, Saraswat M, Kumar S, Pal R, Modwel G (2022). A comprehensive survey of image segmentation: clustering methods, performance parameters, and benchmark datasets. Multimed. Tools Appl..

[17] Zaitoun NM, Aqel MJ (2015). Survey on image segmentation techniques. Procedia Comput. Sci..

[18] Otsu N (1979). A threshold selection method from gray-level histograms. IEEE Trans. Syst. Man Cybern..

[19] Nock R, Nielsen F (2004). Statistical region merging. IEEE Trans. Pattern Anal. Mach. Intell..

[20] Dhanachandra N, Manglem K, Chanu YJ (2015). Image segmentation using k-means clustering algorithm and subtractive clustering algorithm. Procedia Comput. Sci..

[21] Najman L, Schmitt M (1994). Watershed of a continuous function. Signal Process..

[22] Kass M, Witkin A, Terzopoulos D (1988). Snakes: Active contour models. Int. J. Comput. Vis..

[23] Boykov Y, Veksler O, Zabih R (2001). Fast approximate energy minimization via graph cuts. IEEE Trans. Pattern Anal. Mach. Intell..

[24] Plath, N., Toussaint, M. & Nakajima, S. Multi-class image segmentation using conditional random fields and global classification. In: *Proceedings of the 26th Annual International Conference on Machine Learning* 817–824. 10.1145/1553374.1553479 (2009).

[25] Minaee S, Boykov Y, Porikli F, Plaza A, Kehtarnavaz N, Terzopoulos D (2022). Image segmentation using deep learning: A survey. IEEE Trans. Pattern Anal. Mach. Intell..

[26] Cuevas C, Martínez R, García N (2016). Detection of stationary foreground objects: A survey. Comput. Vis. Image Underst..

[27] Berjón D, Cuevas C, Morán F, García N (2018). Real-time nonparametric background subtraction with tracking-based foreground update. Pattern Recognit..

[28] Dulayatrakul, J., Prasertsakul, P., Kondo, T. & Nilkhamhang, I. Robust implementation of hand gesture recognition for remote human–machine interaction. In: *2015 7th International Conference on Information Technology and Electrical Engineering (ICITEE)* 247–252. 10.1109/iciteed.2015.7408950 (IEEE, 2015).

[29] Mandellos NA, Keramitsoglou I, Kiranoudis CT (2011). A background subtraction algorithm for detecting and tracking vehicles. Expert Syst. Appl..

[30] Yao R, Lin G, Xia S, Zhao J, Zhou Y (2020). Video object segmentation and tracking: A survey. ACM Trans. Intell. Syst. Technol. (TIST).

[31] Cuevas C, Quilón D, García N (2020). Automatic soccer field of play registration. Pattern Recognit..

[32] Kataoka, H., Hashimoto, K. & Aoki, Y. Player position estimation by monocular camera for soccer video analysis. In: *SICE Annual Conference 2011* 1985–1990 (2011).

[33] Hoernig, M., Herrmann, M. & Radig, B. Real time soccer field analysis from monocular TV video data. In: *11th International Conference on Pattern Recognition and Image Analysis (PRIA-11-2013)*, vol. 2 567–570 (2013).

[34] Cioppa, A., Deliege, A. & Van Droogenbroeck, M. A bottom-up approach based on semantics for the interpretation of the main camera stream in soccer games. In: *Proceedings of the IEEE Conference on Computer Vision and Pattern Recognition Workshops* 1765–1774. 10.1109/cvprw.2018.00229 (2018).

[35] Hoernig M, Herrmann M, Radig B (2015). Real-time segmentation methods for monocular soccer videos. Pattern Recognit. Image Anal..

[36] Javed A, Malik KM, Irtaza A, Malik H (2020). A decision tree framework for shot classification of field sports videos. J. Supercomput..

[37] Ali, M. M. N., Abdullah-Al-Wadud, M. & Lee, S.-L. An efficient algorithm for detection of soccer ball and players. In: *Proceedings of 16th ASTL Control and Networking*, vol. 16 39–46 (2012).

[38] Rao, U. & Pati, U. C. A novel algorithm for detection of soccer ball and player. In: *IEEE International Conference on Communications and Signal Processing (ICCSP)* 0344–0348. 10.1109/iccsp.2015.7322903 (2015).

[39] Sarkar, S., Chakrabarti, A. & Mukherjee, D. P. Generation of ball possession statistics in soccer using minimum-cost flow network. In: *2019 IEEE/CVF Conference on Computer Vision and Pattern Recognition Workshops (CVPRW)* 2515–2523. 10.1109/cvprw.2019.00307 (2019).

[40] Rianthong, T., Thewsuwan, S., Charoenpong, T. & Pattanaworapan, K. A method for detecting lines on soccer field by color of grass variation. In: *IEEE International Conference on Knowledge and Smart Technology (KST)* 131–134. 10.1109/kst48564.2020.9059550 (2020).

[41] Choroś, K. Automatic playing field detection and dominant color extraction in sports video shots of different view types. In: *Multimedia and Network Information Systems* 39–48. 10.1007/978-3-319-43982-2_4 (2017).

[42] Huang, Y., Llach, J. & Bhagavathy, S. Players and ball detection in soccer videos based on color segmentation and shape analysis. In: *International Workshop on Multimedia Content Analysis and Mining* 416–425. 10.1007/978-3-540-73417-8_50 (2007).

[43] Khatoonabadi SH, Rahmati M (2009). Automatic soccer players tracking in goal scenes by camera motion elimination. Image Vis. Comput..

[44] Quilón, D., Mohedano, R., Cuevas, C. & García, N. Unsupervised high-quality soccer field segmentation. In: *IEEE International Symposium on Consumer Electronics (ISCE)* 1–2. 10.1109/ISCE.2015.7177808 (2015).

[45] Qian, Y. & Lee, D. D. Adaptive field detection and localization in robot soccer. In: *Robot World Cup* 218–229. 10.1007/978-3-319-68792-6_18 (2016).

[46] https://pxhere.com/. [Images under CC0 license].

[47] Zahn, A. *Manchester United Match Postponed Over Dummy Bomb*. https://media.socastsrm.com/wordpress/wp-content/blogs.dir/1930/files/2019/02/Old_Trafford_inside_20060726_1.jpg. [Under CC-BY license] (2016).

[48] Wikipedia user LtPowers: Women’s Professional Soccer—2011 Championship—Flash vs Independence. https://commons.wikimedia.org/wiki/File:Women%27s_Professional_Soccer_-_2011_Championship_-_Flash_vs_Independence.jpg. [Under CC-BY license] (2011).

[49] Akaike H (1974). A new look at the statistical model identification. IEEE Trans. Autom. Control.

[50] Soille P (2004). Morphological Image Analysis: Principles and Applications.

[51] Homayounfar, N., Fidler, S. & Urtasun, R. Sports field localization via deep structured models. In: *Proceedings of the IEEE Conference on Computer Vision and Pattern Recognition* 5212–5220. 10.1109/cvpr.2017.427 (2017).

[52] Kumar, N. P., Satoor, S. & Buck, I. Fast parallel expectation maximization for Gaussian mixture models on gpus using CUDA. In: *IEEE International Conference on High Performance Computing and Communications* 103–109. 10.1109/HPCC.2009.45 (2009).

[53] Wikipedia user Voltmetro: Lionel Messi missed a penalty kick. https://en.wikipedia.org/wiki/2018_FIFA_World_Cup_Group_D#/media/File:FWC_2018_-_Group_D_-_ARG_v_ISL_-_Messi_penalty_kick.jpg. [Under CC-BY license] (2018).

[54] Wikipedia user Kevster: De Euroborg, het stadion van FC Groningen. https://commons.wikimedia.org/wiki/File:Euroborg.jpg. [Under CC-BY license] (2006).

[55] Wikipedia user Rickazio: Stadium of FC Groningen. https://commons.wikimedia.org/wiki/File:EuroborgGroningen2015.jpg. [Under CC-BY license] (2015).

